# ESG Performance and Stock Price Volatility in Public Health Crisis: Evidence from COVID-19 Pandemic

**DOI:** 10.3390/ijerph19010202

**Published:** 2021-12-25

**Authors:** Dongyi Zhou, Rui Zhou

**Affiliations:** School of Economics, Fudan University, Shanghai 200433, China; 17300680139@fudan.edu.cn

**Keywords:** public health crisis, ESG, stock price volatility, avoid risk, COVID-19

## Abstract

Unlike traditional financial crises, COVID-19 is a global public health crisis with a significant negative impact on the global economy. Meanwhile, the stock market has been hit hard, and corporate share prices have become more volatile. However, the stock prices of some enterprises with good performance of ESG (Environment, Social, and Governance) are relatively stable in the epidemic. This paper selects ESG rating data from MSCI (Morgan Stanley Capital International) with better differentiation, adopts multiple regression and dummy variables, and adopts the Differences-in-Differences (DID)model with the help of COVID-19, an exogenous event. Empirical test the impact of ESG performance on the company’s stock price fluctuations. The results show that the stock price volatility of companies with good ESG performance is lower than that of companies with poor performance. Second, COVID-19 exacerbates volatility in company stock prices, but the increase in stock price volatility of companies with good ESG performance is small. That is, good ESG performance helps reduce the increase in stock price volatility due to COVID-19 shock, and plays a role in enhancing “resilience” and stabilizing stock prices. This paper provides new empirical evidence for the study of ESG performance and corporate stock price volatility, and puts forward relevant policy recommendations for enterprises and government departments.

## 1. Introduction

In the course of the financial crisis, investors and scholars have paid extensive attention to what type of company’s stock price performed better or alleviated the systemic risk. Many scholars have carried out in-depth discussions. The research found that companies with good ESG performance had a strong slow-release effect on systemic risk [1,2,3,4,5,6,7,8]. Especially during the COVID-19 pandemic, relevant studies have reached a new climax. Most studies show that ESG has obvious “resilience” and good avoid risk effect in crisis, thus effectively reducing systemic risk [9,10,11].

The sudden outbreak of COVID-19 made the world suffer a public health crisis as well as a heavy economic blow. After a short break during the Spring Festival in 2020, China’s stock market experienced a “1000 share limit drop”, with the CSI 300 index plummeting from 4200 to 3700 points, and the fluctuation of enterprise stock prices increased under the impact of the epidemic. This raises new questions about what types of stocks perform better in a crisis and are more resistant to systemic financial risk. What role does ESG performance play in stabilizing stock prices and reducing volatility? Can the improvement of ESG’s performance become a “cushion” for enterprises in a crisis? What will be the effect on investors? These questions are worthy of our study.

The purpose of this study is to explore the relationship between ESG performance and stock price volatility under COVID-19. The research is divided into three levels: first, linear regression is used to test the effect of ESG performance on corporate volatility during COVID-19; Then, referring to the research ideas of Diaz et al. [12], the samples were stratified according to ESG score, and the groups of companies with better ESG performance and those with poor ESG performance were selected. Dummy variables were set to explore the volatility difference between the groups, so as to verify whether the volatility of companies with better ESG performance was significantly lower than that of companies with poor ESG performance. Finally, considering the impact of the COVID-19 outbreak and using the research ideas of Hoepner and Oikonomou [13] for reference, difference-in-differences (DID for short) are used to explore the impact of the COVID-19 outbreak. Does ESG performance enhance the company’s “resilience”?

The innovation of this paper lies in: (1) the research perspective is novel. From the perspective of COVID-19, this paper studies the relationship between ESG performance and stock price volatility of enterprises under the impact of COVID-19. Most existing studies focus on the relationship between ESG performance, corporate performance and enterprise value, while this paper focuses on the effect of ESG performance on stock price volatility of enterprises. (2) High data quality. Existing literature is mostly used at present in China “picture of melt green” institutions such as disclosure of ESG rating data. However, these data are the enterprise of ESG performance roughly divided into several levels, A+ to D and for companies at the same level of ESG, the marking and no difference, so this method is divided into A rough, poor differentiation. The data used in this paper are MSCI ESG rating scores disclosed by Bloomberg data terminal, which quantifies enterprise ESG performance into floating point numbers with higher accuracy and better differentiation. (3) Use of new econometric methods. Most of the literature related to ESG used the multivariate linear regression method. This paper treats the outbreak of COVID-19 pandemic as a quasi-natural experiment and studies the effect of ESG performance on the share price volatility in the public health crisis using the difference-in-difference method (DID).

The following structure of this paper is as follows: The second part is a literature review, which reviews the concept and development of CSR and ESG, and summarizes the impact of COVID-19 on the stock market and the avoiding risk role of ESG. The third part is research design, including data source, variable selection and econometric model construction. The fourth part is the analysis of test results, including descriptive statistics, baseline regression, robustness test; The fifth part is the conclusion and enlightenment.

## 2. Literature Review

### 2.1. Corporate Social Responsibility (CSR) and Environmental Social Governance (ESG)

Corporate social responsibility (CSR) refers to the responsibility of industrial and commercial enterprises to not only be responsible to shareholders but also meet the expectations of different stakeholders in society when carrying out business activities [14]. ESG is an acronym developed by 20 financial institutions in response to the appeal of UN Secretary-General Kofi Anon in a 2004 report, which represents three different dimensions of Environment, Social responsibility and corporate Governance. It is the main evaluation method to inspect whether the enterprise operation conforms to sustainable development, and it is also the extension of the concept of green finance. Generally speaking, CSR refers to the activities of a company in promoting social responsibility and becoming a better corporate citizen. ESG refers to how companies and investors integrate environmental, social and governance issues into their business models. The difference between the two terms is that ESG explicitly encompasses governance, while CSR indirectly encompasses governance issues, as both involve environmental and social factors. Therefore, ESG is a broader concept than CSR [15].

North America and Europe are world leaders in CSR practices. After years of practice, a number of sustainable development frameworks and related standards have been put forward. At present, the mainstream enterprise sustainable development frameworks and related standards include SA8000, UNGC, G4-GRI and ISO 26000. Meanwhile, ESG related investment strategies develop rapidly and have gradually evolved into one of the mainstream investment strategies. In contrast, China’s practice and related theoretical research started late and is limited in scale. The research of Chandan and Das [16] shows that the state-centric corporate social responsibility (CSR) model is different from the market model in the United States and the relationship model in the European Union. Hou and Li [17] believe that the differences in the development of CSR in China, Europe and the United States are caused by history, economy and enterprise development. ESG’s domestic development in China is still not fully mature. In developed countries, institutional investors play a very important role in ESG investment practice. Institutional investors continually monitor ESG performance as they build their portfolios. In China, institutional investors exist but are not mainstream, and most investors are still retail investors. Accordingly, the demand for ESG products is still relatively small, and investors are more inclined to consider the factor of ESG performance on a psychological level, rather than systematically including it into the scope of investigation when allocating asset portfolios in a quantitative form [9].

### 2.2. The Impact of COVID-19 on China’s Stock Market

In January 2020, COVID-19 was detected in Wuhan, China. Within a month, the number of cases in Wuhan soared to 60,000, leading to a total “lockdown” in the city and much of China. The virus brought about an unprecedented global public health crisis, followed by a global market-wide financial crisis. At 10 am on 23 January, Wuhan was locked down and the stock market was closed for the Lunar New Year. China’s stock markets reacted to the public health crisis shortly after the lockdown of Wuhan. On 3 February 2020, the market reopened and the CSI 300 index fell sharply from 4200 to 3700. In general, the epidemic has brought a great negative impact on the stock market. To be specific, the study by Baker et al. [18] shows that in the 22 trading days from 24 February to 24 March 2020, 18 markets had a fluctuation of 2.5% or more every day. COVID-19 has affected policymakers, investors and companies across the globe. Dayong et al. [19] found that COVID-19 has caused markets to become highly volatile and unpredictable. Duan et al. [20] discovered that stock returns and turnover rates were positively predicted by the COVID-19 sentiments during the period from 17 December 2019 to 13 March 2020, which illustrated how the effects of the pandemic crisis were amplified by the sentiments. Further research found that COVID-19 had an unprecedented negative impact on stock market volatility compared to the impact of other epidemics. Duan et al. [21] develop two COVID-19 sentiment indices that capture the moods related to COVID-19. Our sentiment indices are real-time and forward-looking indices in the stock market. We discover that stock returns and turnover rates were positively predicted by the COVID-19 sentiments during the period from December 17, 2019 to March 13, 2020. Hanif et al. [22] studied the spillover effect of the stock market and found that COVID-19 increased the risk spillover effect of the market between March 2020 and April 2020. Huang et al. [23] found that the stock price crash risk of energy firms significantly decreases in the post-COVID-19 period and the effect of COVID-19 on stock price crash risk is less severe for state-owned enterprises (SOEs) than for non-SOEs in the post-COVID-19 period.

### 2.3. Avoid Risk Role of ESG

#### 2.3.1. ESG and Risk

There is a view that the risk exposure of a company is related to ESG status. Heinkel et al. [1] established a model and divided the capital market according to investors’ preferences. The study believed that companies with high ESG scores were given higher valuations and suffered lower systemic risks. Chen et al. [2] verified the inhibitory effect of CSR/ESG performance on the downside risk of stock price by describing the asymmetry of stock return distribution. Mishra and Modi [3] use empirical analysis to verify that good corporate social responsibility can help reduce non-systemic risks. The empirical study of Diemont, et al. [4] shows that tail risk measurement is closely related to ESG risk. The research results of Sassen [24] confirm that the improvement of ESG performance has a prominent effect on reducing stock price volatility and eliminating the overall risk of the company. Garcia et al. [5] measured the relationship between ESG performance and systemic risk by analyzing the performance of 365 companies from 2010 to 2012, and verified that the relationship between ESG performance and systemic risk was in an inverted “U” shape. The study of Jagannathan and Ravikumar [6] found that ESG-related risks may be rare, huge and non-diversified, especially related to the downside risks of enterprises. According to the research results of Albuquerque and Koskinen [8], when a company increases product differentiation through social responsibility investment, the higher the social responsibility investment, the lower the systemic risk of the company and the higher the value of the company. Lueg and Krastev [7] studied the two-way influence of ESG disclosure and corporate risk, and the results show that high-quality disclosure of sustainable development performance is conducive to corporate risk reduction. Ilhan et al. [25] found that companies with poorer ESG performance had higher tail risks. The research of Hoepner et al. [13] shows that companies that take an active role in ESG/CSR, especially those that attach importance to the environment, can reduce their downward risks in a crisis. Shakil [26] finds a significant adverse influence of ESG on stock price volatility. However, firm size portrays a non-significant moderating effect on ESG-stock price volatility nexus. And portfolio managers may invest in high achieving ESG firms to leverage the market volatility of their portfolio. Sabbaghi [27] using the Morgan Stanley Capital International (MSCI) indices as proxies for ESG test assets, this study investigates volatility risk for the highest ESG-rated firms through an empirical analysis in assessing how good news and bad news impact the risk of ESG firms. The analysis provides empirical evidence in support of the hypothesis that the impact of news on the volatility of ESG firms is larger for bad news, compared to good news. Employing an EGARCH framework, the analysis also finds that, in response to bad news, the observed volatility increases for small size ESG firms is lower compared to large and mid-cap ESG firms. James [28] also found that stocks with low ESG risk ratings (green stocks) not only have higher realized returns but also provide better tail-risk protection than stocks with high ESG risk ratings (brown stocks), especially during the COVID-19 crisis. The tail-risk protection provided by green stocks is robust within sectors and styles. Green funds and exchange-traded funds (ETFs) that hold green stocks have attracted significantly more fund flow than their counterparts, which is associated with the outperformance for both green funds and stocks.

#### 2.3.2. The Avoid Risk Role of ESG in Crisis

Benabou and Tirole [29] found that companies with different ESG and CSR performance may face different systemic risk exposures, because they are resilient and have the ability to recover faster from crises. Oikonomou et al. [30] used downside risk as a measurement index. The author conducted an empirical study based on the S&P 500 index from 1992 to 2009, and the results showed that corporate social responsibility was negatively correlated with financial risk. Krueger [31] believes that companies with better ESG performance can be more “resilient” and recover from shocks more quickly in the face of negative events unique to the company. Lins et al. [32] found that companies with high ESG scores outperformed those with low ESG scores during the 2008–2009 financial crisis. According to Zhang [19], strong ESG/CSR companies face relatively small price elasticity demand due to product differentiation strategy, thus reducing system risk. Broadstock, et al. [9] explored that the performance of companies with high ESG score was better than that of companies with low ESG score in the context of COVID-19, and the event study method was used to prove that ESG played a avoid risk role to a certain extent in the crisis period compared with the normal period. Albuquerque et al. [10] used differential difference method to measure the mitigation effect of ESG input on corporate downside risk during the crisis, and the results showed that the downside risk of companies with excellent ESG performance was significantly lower than that of companies with poor ESG performance. The research of Diaz et al. [12] shows that environment and society are the main dimensions that can reflect the role of avoiding risk during the COVID-19 crisis. Takahashi and Yamada [11] Identify factors affecting the Japanese Stock market during the COVID-19 pandemic period. Studies have shown that governance (ESG) engagement, there is no evidence that firms that have highly rated ESG scores have higher abnormal returns, but firms with ESG funds outperform those without.

Albuquerque et al. [10] selected data during the COVID-19 epidemic to verify that companies with high ES scores in the ESG scoring system showed lower stock price volatility in the first quarter of 2020. However, it should be noted that this paper is significantly different from Albuquerque et al. [10] Albuquerque et al. [10] focuses on the impact of ES (environmental and social responsibility) performance on company stock price performance. This paper focuses on the “buffer” effect of ESG performance on company stock price volatility under the impact of the epidemic. Albuquerque et al. [10] used the main index ES is environment (E) and social responsibility (S), ignoring corporate governance (G). The data in this paper are the MSCI ESG rating score disclosed by Bloomberg data terminal, which quantifies the ESG performance of enterprises into floating point numbers. Other advantages are higher accuracy and better differentiation. In addition, China’s ESG investment concept was formed late. A-shares were officially included in the MSCI index in 2018. A-share listed companies were selected as samples in this paper, which contributed Chinese experience to ESG related research.

To sum up, ESG related research is a relatively emerging research topic in recent years, and since performance and income related data are easier to obtain and more frequent, most research focuses on the effect of ESG on corporate performance. In recent years, due to the improvement of ESG information disclosure system, the improvement of data availability and the occurrence of several financial crises, people began to discuss the relationship between ESG and risk under the impact of financial crises. Most of the existing literature on ESG performance and corporate risk in the academic world is to explore the relationship between ESG performance and stock price fluctuations of a company. However, these studies mainly focus on the period of stable economic operation, and seldom consider the “impact” of economic crisis. However, in the few literature that consider the effect of ESG performance on corporate risk under crisis, most of them only focus on ESG performance during the financial crisis, without comparison before and after the crisis. In addition, there is little literature on the impact of financial crises resulting from public health events on financial markets. Therefore, it is of great theoretical and practical significance to further study the avoided risk effect of ESG before and after the impact of the crisis, especially the mitigation effect of ESG on systemic risk under the impact of COVID-19, a public health crisis different from the financial crisis.

## 3. Study Design

### 3.1. Data Source

At present, MSCI, FTSE, Thomson Reuters, Morningstar and Sustainalytics are the major ESG rating agencies and systems in the world. In China, there are different ESG scoring matrices given by rating systems such as China Securities Index, Shangdao Ronggreen and Social Investment Alliance. Currently, the available data is mainly based on rough grade classification, while THE MSCI ESG index further discloses the specific score of each dimension of E, S and G on the basis of grade classification, ranging from the minimum 0.1 to the maximum 100, thus providing a complete ESG measure. In combination with the availability of data, this paper quantifies ESG performance in the MSCI index disclosed in Bloomberg database to measure different corporate governance, environmental and social performance of A-share listed companies.

This paper selects listed companies in the A-share market as samples, and the sample range is all trading days from 1 December 2019 to 31 March 2020. At the same time, the following three samples of ST companies, data missing and MCSI’s ESG rating system are excluded. The ESG performance score is derived from the MSCI ESG index of Bloomberg Data Terminal. Tobin’s Q value came from CSMAR database. Volatility, high price, low price, average price and other corporate financial indicators come from the Wind financial terminal. Finally, valid data for 1021 companies were obtained.

The ESG performance score is derived from the MSCI ESG index of Bloomberg Data Terminal. Tobin’s Q value came from CSMAR database. Volatility, high price, low price, average price and other corporate financial indicators come from the Wind financial terminal. The cleaning, screening and organization of the original data were completed by Microsoft Excel software, and the regression was completed by Stata software.

### 3.2. Variables Selection

#### 3.2.1. Explained Variable: Volatility

Volatility in this paper is divided into window volatility and intraday volatility. Window period volatility refers to the day after Wuhan was closed down, namely 24 January 2020, as the base date. Different days are set before and after the base date to form window periods covering different time ranges. The calculation formula of window yield is as follows:

$$VOL_{i}=\sqrt{\frac{\sum_{t=1}^{N}\left[{\left(R_{it}-\frac{\sum_{t=1}^{N}R_{it}}{N}\right)}^{2}\right]}{N-1}}\;R_{it}=\frac{P_{it}}{P_{i,t-1}}-1$$

where, $P_{it}$ is the closing price of stock I on day *t*, and *N* is the window period.

Daily stock prices within the window period are selected to calculate the volatility within this range, which is recorded as window period volatility. The window periods selected in this paper include 5 trading days before and after the base date, 10 trading days before and after the base date, and the first quarter of 2020. Intraday volatility is the intraday high, low and average price of all trading days between 1 November 2019 and 31 March 2020. The results calculated by using these three groups of data can approximately replace intraday volatility, and the interest calculation formula is as follows:

(1) $$vol_{it}=(High_{it}-Low_{it})/Avg_{it}$$

In Formula (1), $vol_{it}$ is the intra-day volatility of company *i* on date *t*, $High_{it}$ is the intra-day high price of company *i* on date *t*, $Low_{it}$ is the intra-day low price of company *i* on date *t*, $Avg_{it}$ is the intra-day average price of company *i* on date *t*. The calculation reflects the maximum daily volatility of a company’s stock. We refer to Parkinson [33], Garman and Klass [34], Rogers and Satchell [35], and Yang and Zhang [36]. But we try to study this problem from another angle. This paper pays more attention to the protection and buffer role of ESG performance when the company’s stock price fluctuates violently under the impact of the epidemic. In view of the fact that COVID-19 is rapid and has a wide range of impact and is exogenous to enterprises, ESG performance can be divided into processing group and control group based on company characteristics. Therefore, the double difference method is very suitable for this study, and it can identify COVID-19’s impact on stock price volatility and the difference of price fluctuation between companies with different ESG performance.

The higher the volatility, the more severe the volatility of financial asset prices, the higher the uncertainty of asset returns. On the contrary, the smaller the volatility, the gentler the price change of financial assets. Volatility in this paper is mainly used to measure risk.

#### 3.2.2. Core Explanatory Variable: ESG

In China, ESG score data is mainly measured by MSCI ESG Index, FTSE Russell ESG Index, China Securities Index, Business Green Index, Social Value Investment Alliance Index and other rating systems.

In the three dimensions of ESG, Governance (G), also known as corporate governance, is the most important, because corporate Governance risks are common and critical to all companies. Compared with corporate governance, Environment (E) and Social (S), namely, Environment and society, are endowed with different degrees of importance in different industries. In the two dimensions of the environment and society, due to the rise of the concept of “climate assistance” in recent years, the discussion on the environmental dimension has become more intense.

The ESG rating system is designed to measure a company’s resilience to significant environmental, social and governance risks to the industry over the long term. Taking the MSCI ESG rating used in this paper as an example, the establishment method of ESG scoring matrix is as follows: The first step is to select 35 indicators of different dimensions from the three levels of environment, society and corporate governance. The second step is to look at how companies perform on each of the 35 different indicators and give them a score for their performance. The third step is to calculate the weighted average of the 35 scores according to MSCI’s weighting method as the total score of ESG. The last step is to adjust the ESG score of the enterprise into seven grades from “AAA” to “CCC”. Among them, AAA and AA mark the leading companies in the industry, A, BBB and BB mark the average company in the industry, and B and CCC mark the backward company in the industry. Other ESG rating systems work in a similar way, assigning certain points to different companies’ performance in environmental, social and corporate governance aspects, and quantifying them into an overall score through weighted average. The difference between different systems mostly lies in the selection of indicators of different dimensions and different weights.

As most of the available ESG data are roughly classified, the MSCI ESG index disclosed by Bloomberg platform further discloses the specific scores of E, S and G on the basis of the classification, ranging from the minimum 0.1 to the maximum 100, thus providing a complete ESG measure. Therefore, this paper selects the TOTAL ESG score data of more than 1000 A-share listed companies covered by THE MSCI ESG rating system, and the higher the ESG score, the better the company’s performance in CSR.

#### 3.2.3. Control Variables

In this paper, specific characteristics of enterprises and industries are controlled. Referring to the research of Broadstock et al. [9], enterprise Size (Size), financial leverage (Lev), TobinQ and Cash holding ratio (Cash) are selected as enterprise characteristic variables to control. On this basis, Fixed effects of control industry. Sabbaghi [27] found that when negative news occurs, the volatility of stock prices of large-sized enterprises also increases significantly. However, Shakil [26] found in his study that enterprise size had no significant moderating effect on the relationship between ESG and stock price volatility. Therefore, enterprise Size may also affect stock price fluctuations, so we add enterprise Size into the control variable. The size of the enterprise is expressed in this paper by the natural logarithm of the total market value of the company, which is the data disclosed in the 2019 annual report of the company. Industry division is based on the CSRC industry code disclosed by Wind terminal. Variable selection and definition are shown in Table 1.

### 3.3. Econometric Model

The empirical test in this paper will be divided into three levels, and three models will be used to verify them respectively.

First, establish the regression equation between ESG performance and the company’s volatility during COVID-19:

(2) $$VOL_{i}=\alpha_{0}+\alpha_{1}ESG_{i}+\alpha_{2}Size_{i}+\alpha_{3}Lev_{i}+\alpha_{4}TobinQ_{i}+\alpha_{5}Cash_{i}+\varepsilon_{i}$$

In Formula (2), VOL represents volatility in the window period: 5 trading days before and after the base date, 10 trading days, and the first quarter of 2020 are successively selected, and the volatility in these three window periods are respectively denoted as VOL5, VOL10 and VOLQ. $\alpha_{0}$ is a constant term, $\varepsilon_{it}$ is a residual term, and $\alpha_{1}$ represents the degree of effect of ESG performance on volatility. If $\alpha_{1}$ is significantly negative, it verifies that good ESG performance is conducive to reducing the volatility of asset prices, making asset prices more stable and thus reducing risks.

Secondly, on the basis of Formula (2), if the company is divided into the group with better ESG performance and the group with poor ESG performance, whether the volatility of the former group will be significantly lower in the same period compared with the latter group. This paper introduces a dummy variable as an ESG factor to distinguish different groups. Therefore, the regression model for the comparison of volatility between companies with excellent ESG performance and those with poor ESG performance during COVID-19 is constructed:

(3) $$VOL_{i}=\alpha_{0}+\alpha_{1}ESGf_{i}+\alpha_{2}Size_{i}+\alpha_{3}Lev_{i}+\alpha_{4}TobinQ_{i}+\alpha_{5}Cash_{i}+\varepsilon_{i}$$

In Formula (3), ESGf is the dummy variable. The processing of this variable is to sort the ESG scores of all companies from large to small and divide them into four equal parts, extract the top 25% and the bottom 25% of companies, assign the value of the top 25% to 1, and the ESGf value of the bottom 25% to 0. Thus, companies with excellent ESG performance can be distinguished from those with poor ESG performance. The other assumptions are the same. The dependent variable is volatility in the window period, VOL.

Finally, by comparing the difference between the control group and the experimental group before and after the implementation of quasi-natural experiment, the method constructs the difference statistics reflecting the implementation effect of quasi-natural experiment. At the same time, this statistic can also reflect the enhancement or alleviation of experimental implementation effect by dividing treatment groups and the basis of illumination. Because the impact of COVID-19 is global, all businesses are affected almost simultaneously. Therefore, the control group and the treatment group were constructed from the perspective of enterprise characteristics by referring to many literatures [37]. In this paper, the treatment group and the control group were divided according to the ESG score of enterprises. That is, the virtual variable ESGf in Model (2) was directly used as the grouped virtual variable.

The DID method was used to study whether companies with excellent ESG performance had stronger “resilience” after the outbreak compared with those with poor ESG performance before the outbreak, that is, whether they could recover from the shock more quickly. Therefore, in order to characterize the effect of ESG performance on corporate volatility before and after COVID-19, the following regression equation is constructed in this paper:

(4) $$vol_{it}=\beta_{0}+\beta_{1}ESGf_{it}+\beta_{2}Post_{it}+\beta_{3}ESGf_{it}\times Post_{it}+\beta_{4}Size_{it}\;+\beta_{5}Lev_{it}+\beta_{6}TobinQ_{it}+\beta_{7}Cash_{it}+\varepsilon_{it}$$

In Formula (4), the time span is selected from trading days between 8 January 2020 and 19 February 2020. The dependent variable is intraday volatility, vol. The sample companies also select the 25% with the highest ESG score and the 25% with the lowest ESG score, a total of 531 companies. Set ESGf and Post dummy variables and their interaction item ESG∗Post. The meaning and treatment of ESGf are the same as above; Post is a time dummy variable used to distinguish between before and after the epidemic impact. The division is based on the closing day of Wuhan, China on 23 January 2020. This variable in the data of subsequent trading days is assigned as 1, and the previous data is assigned as 0.

## 4. Analysis of Inspection Results

### 4.1. Descriptive Statistics

Table 2 lists the sample size, mean, standard value, minimum value and maximum value of each variable. As the intraday volatility covers multiple dates and is panel data, there are many observed values.

### 4.2. Benchmark Regression

#### 4.2.1. Volatility between ESG and COVID-19

As can be seen from the regression results in Table 3, excellent ESG performance of listed companies contributes to the stability of the company’s stock price. Since the coefficient $\alpha_{1}$ before ESG is significantly negative, it can be inferred that, for a firm, the higher the ESG score, the lower its volatility over time. As window volatility is selected for 5 trading days before and after the base date, 10 trading days, and the volatility for the whole first quarter of 2020, ESG coefficients α_1 in columns (1), (3) and (5) of Table 3 are all significantly negative, so this conclusion is robust. In addition, Table 3 (2), (4) and (6) are listed as the results of the fixed effect model regression. Controlling the industry fixed effect can solve the endogeneity problem caused by the missing variables to a certain extent. The ESG coefficient α_1 is significantly negative, being −0.127, −0.145 and −0.132 respectively, indicating that when the ESG score increases by 1, stock price volatility decreases by 0.127, 0.145 and 0.132 over 5 trading days, 10 trading days, and the first quarter of 2020.

#### 4.2.2. Comparison of Volatility of ESG Performance during COVID-19

Further, we analyze and compare the COVID-19 volatility between high performing and low performing ESG companies. As can be seen from the regression results in Table 4, companies with excellent ESG performance have higher avoid risk ability compared with companies with poor ESG performance, and their volatility is significantly lower than that of companies with poor ESG performance. As the coefficient before ESG is significantly negative, which is −0.044, −0.046 and −0.047 respectively, that is, the stock price volatility of companies with good ESG performance is 0.044, 0.046 and 0.067 lower than that of companies with average ESG performance in the 5 trading days and 10 trading days before and after the base date of the company and in the first quarter of 2020. This shows that companies with good ESG performance have a strong ability to resist risks.

#### 4.2.3. The Effect of ESG on Corporate Volatility before and after COVID-19

(1) Parallel trend test

**Figure 1:**
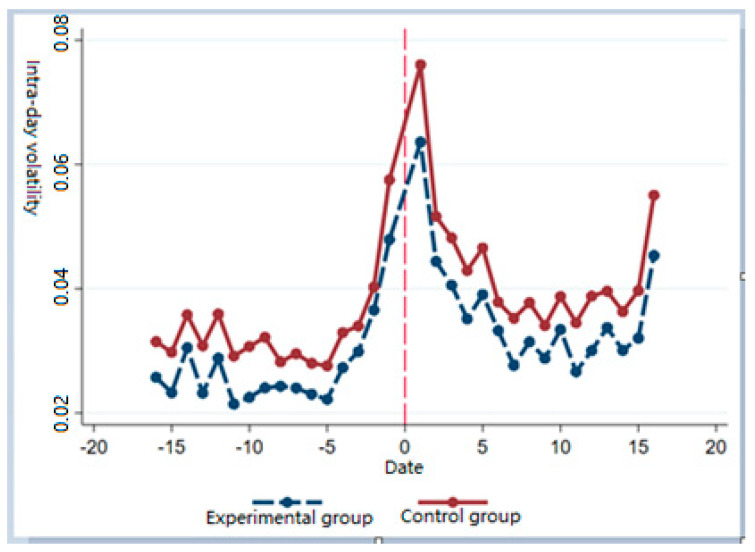
Intra-day volatility trend of the experimental and control groups before and after COVID-19 outbreak.

Figure 1 shows the intra-group mean changes of intraday volatility in the experimental group (companies with high ESG performance) and the control group (companies with low ESG performance) before and after the outbreak. The results showed that the variation trend of intraday volatility of the experimental group and the control group before the epidemic impact was basically the same, meeting the parallel trend test, and the intraday volatility of the experimental group was always lower than the control group, which was consistent with the above analysis.

(2) Regression analysis

The regression results of the impact of ESG on the company’s stock price volatility before and after COVID-19 are shown in Table 5. As can be seen from Table 5, since the time dummy variable represents the time limit of the impact of COVID-19, and the former coefficient is significantly positive, this shows that under the impact of COVID-19, the stock price volatility of enterprises has increased and the degree of instability has increased. The coefficient $\beta_{1}$ before the policy dummy variable ESGf is significantly negative, this indicates that the stock price volatility of companies with excellent ESG performance is lower than that of companies with poor ESG performance. The coefficient $\beta_{3}$ before interaction term is significantly negative, indicating that after excluding other impacts of COVID-19, the increase of volatility of companies with excellent ESG performance is 0.002 lower than that of companies with poor ESG performance. This phenomenon can be seen as an illustration of the “weakening” of ESG performance against shocks. In the crisis, ESG performance becomes the “cushion” of the company, enabling the company to have stronger “resilience” and recover from shocks faster.

In general, we consider ESG investment as a kind of good deed to society and a cost investment made by enterprises at the expense of economic resources. However, the research results of this paper have obvious economic significance. The research results reveal the negative correlation between ESG performance and stock price volatility, thus helping enterprises and investors to understand the benefits of ESG input more comprehensively. On the one hand, ESG performance can help companies achieve more robust stock price performance. On the other hand, it also helps investors better manage risks and build portfolios based on ESG factors.

### 4.3. Robustness Test

#### 4.3.1. Change the Subsample Selection Method

As for the second regression model Formula (3) above, the classification of ESG factor is based on the 25% companies with the highest ESG score and the 25% with the lowest ESG score. As the two groups of companies with the best performance and the worst performance are selected as sub-samples, the regression results may be affected by this selection method. Therefore, we changed the selected sub-sample, adjusted the combination of the highest 25% and the lowest 25% ESG score to the highest 25% and 50–75% ESG score companies, and assigned the value ESGf of the top 25% companies to 1, and the value ESGf of the 50–75% companies to 0, and then regression. The regression results are shown in Table 6.

As can be seen from Table 6, when the volatility in the window period is selected for 10 trading days before and after the base day and the first quarter of 2020, the regression result is still significantly negative; while when the volatility in the window period is selected for 5 days before and after the base day, the regression effect is no longer significant. The reason for this result may be that it takes a period of time for ESG performance to show its weakening effect on volatility. Compared with the short term, the weakening effect will be more obvious after a period of time. Therefore, on the whole, the model is robust.

#### 4.3.2. Change the Time Span

As the third econometric model Formula (4) mentioned above may be affected by the number of periods included in the time span before and after COVID-19 impact when it is tested by DID method. Therefore, this paper adjusts the time span, extending the trading daytime span from 8 January 2020 to 19 February 2020 to 2 December 2019 to 31 March 2020, and then makes a regression according to the model ideas mentioned above. The test results are shown in Table 7, which shows that the results are still robust.

## 5. Conclusions

From the perspective of COVID-19 shock, this paper selects THE MSCI ESG data disclosed by Bloomberg data terminal and uses the difference in difference (DID) method to test the relationship between ESG performance and stock price volatility of enterprises under COVID-19 shock. The results show that: (1) excellent ESG performance of single listed companies is conducive to reducing stock price volatility under the impact of crisis and stabilizing stock price; (2) Compared with companies with poor ESG performance, companies with excellent ESG performance had lower volatility and more stable stock prices in the same period of time; (3) Volatility of listed companies generally rose before and after the impact of the epidemic. In this process, companies with excellent ESG performance saw a lower increase in volatility than those with poor ESG performance, showing stronger “resilience” and the ability to recover faster from the impact of the crisis. As a result, ESG performance can act as a “cushion” for a company and serve as a good hedge in a crisis.

At the same time, the research of this paper also gives us the following enlightenment: From an investor’s perspective, investors should factor ESG into their asset allocation considerations and avoid companies with low ESG scores, thereby avoiding costly risks. From the perspective of enterprises, companies with good ESG performance have relatively low stock price volatility. Therefore, enterprises should take an active role in ESG, integrate the concept of corporate social responsibility into the operation system, and create “ESG reputation” for enterprises. From the perspective of policy makers, since ESG performance can reduce the systemic risk of enterprises, relevant policy departments should strengthen support for green enterprises and related projects and encourage participation in the construction of green projects. The government can adopt top-down measures to escort companies with good CSR performance.

## Figures and Tables

**Table 1:** Variable selection and definition.

Variable Types	Variable Name	Variable Code	Variable Definitions
Explained variable	5 days before and after the fluctuations	VOL5	Five (10) days of stock price volatility before and after the window period
	10 days before and after the fluctuation	VOL10	Stock price volatility of 10 days before and after the window period (20 days in total)
	Seasonal fluctuations	VOLQ	Stock price volatility for all trading days in the first quarter of 2020
	Intraday volatility	vol	(Intraday high price—intraday low price)/daytime average price
Explanatory variables	ESG performance	ESG	Quantitative scores given by the MSCI ESG index
Control variables	The enterprise scale	Size	The natural log of a company’s total annual market value
	Financial leverage	Lev	Average annual total liabilities/Average annual Total assets ×100
	Tobin Q value	TobinQ	Company market capitalization/total assets
	Cash holding ratio	Cash	The company holds cash/total assets

**Table 2:** Descriptive Statistics.

Variable Name	Variable Code	Sample Size	Mean Value	Standard Deviation	Min	Max
5-Day Fluctuation	VOL5	1021	2.020	0.222	1.079	2.807
Fluctuation In the Last 10 Days	VOL10	1021	1.818	0.219	1.002	2.714
Quarterly Fluctuation	VOLQ	1021	1.743	0.241	1.029	2.534
Intraday Fluctuation	vol	38,690	0.2294	0.4204	0	1
Esg Performance	ESG	1021	24.80	7.650	11.21	61.72
Tobin Q Value	TobinQ	1021	1.693	1.313	0.730	14.09
Enterprise Scale	Size	1021	23.54	1.159	20.62	28.09
Cash Ratio	Cash	1021	0.0018	0.0282	0	0.876
Financial Leverage	Lev	1021	51.51	21.72	0.836	229.0

**Table 3:** Regression results of ESG and corporate volatility during COVID-19.

	(1)	(2)	(3)	(4)	(5)	(6)
	VOL5	VOL5	VOL10	VOL10	VOLQ	VOLQ
ESG	−0.099 **	−0.127 **	−0.121 ***	−0.145 ***	−0.126 **	−0.132 **
	(0.047)	(0.051)	(0.046)	(0.050)	(0.051)	(0.054)
Size	−0.001	−0.018 ***	0.007	−0.009 *	0.032 ***	0.012 **
	(0.006)	(0.006)	(0.006)	(0.005)	(0.006)	(0.006)
Lev	−0.002 ***	−0.001 ***	−0.001 ***	−0.000	−0.000	0.001
	(0.000)	(0.000)	(0.000)	(0.000)	(0.000)	(0.000)
TobinQ	−0.031 ***	−0.017 ***	−0.034 ***	−0.020 ***	−0.040***	−0.027 ***
	(0.006)	(0.007)	(0.006)	(0.006)	(0.007)	(0.007)
Cash	−0.598 **	−0.493 **	−0.624 ***	−0.541 **	−0.790 ***	−0.698 ***
	(0.237)	(0.224)	(0.235)	(0.221)	(0.258)	(0.238)
Constant term	2.901 ***	2.574 ***	2.725 ***	2.406 ***	2.696 ***	2.383 ***
	(0.141)	(0.144)	(0.140)	(0.142)	(0.153)	(0.153)
Sample size	1021	1021	1021	1021	1021	1021
$R^{2}$	0.100	0.233	0.091	0.238	0.095	0.265
Fixed effects		YES		YES		YES

Note: *, ** and *** represent significant at the level of 10%, 5% and 1% respectively.

**Table 4:** Regression results of the volatility of companies with high and low ESG performance during COVID-19.

	(1)	(2)	(3)
	VOL5	VOL10	VOLQ
ESGf	−0.044 **	−0.046 **	−0.067 ***
	(0.021)	(0.020)	(0.022)
Size	−0.030 ***	−0.033 ***	−0.027 ***
	(0.009)	(0.009)	(0.009)
Lev	−0.001 *	−0.000	0.001
	(0.001)	(0.001)	(0.001)
TobinQ	−0.007	0.000	0.012
	(0.008)	(0.008)	(0.009)
Constant term	2.810 ***	2.628 ***	2.349 ***
	(0.199)	(0.197)	(0.208)
Sample size	526	526	526
$R^{2}$	0.248	0.257	0.292
Fixed effects	YES	YES	YES

Note: *, ** and *** represent significant at the level of 10%, 5% and 1% respectively.

**Table 5:** Regression results of ESG’s effect on corporate volatility before and after COVID-19.

	(1)	(2)
	VOL	VOL
Post	0.011 ***	0.011 ***
	(0.001)	(0.001)
ESGf	−0.005 ***	−0.002 ***
	(0.001)	(0.001)
ESGf*Post	−0.002 ***	−0.002 ***
	(0.001)	(0.001)
Size		−0.001 ***
		(0.000)
Lev		−0.000 ***
		(0.000)
TobinQ		0.002 ***
		(0.000)
Cash		−0.025 ***
		(0.005)
Constant term	0.033 ***	0.063 ***
	(0.000)	(0.004)
*N*	13,247	13,247
*R* ^2^	0.059	0.085

Note: *, ** and *** represent significant at the level of 10%, 5% and 1% respectively.

**Table 6:** Change the subsample selection method.

	(1)	(2)	(3)
	VOL5	VOL10	VOLQ
ESGf	−0.028	−0.040 **	−0.048 **
	(0.020)	(0.020)	(0.021)
Size	−0.029 ***	−0.030 ***	−0.032 ***
	(0.009)	(0.008)	(0.009)
Lev	−0.000	0.001	0.002 **
	(0.001)	(0.001)	(0.001)
TobinQ	−0.009	0.001	0.018 *
	(0.009)	(0.009)	(0.009)
Cash	−0.445 *	−0.494 **	−0.609 **
	(0.237)	(0.230)	(0.244)
Constant term	2.737 ***	2.503 ***	2.388 ***
	(0.197)	(0.191)	(0.203)
Sample size	526	526	526
$R^{2}$	0.257	0.277	0.315
Fixed effects	YES	YES	YES

Note: *, ** and *** represent significant at the level of 10%, 5% and 1% respectively.

**Table 7:** Change the time span.

	(1)	(2)
	VOL	VOL
Post	0.015 ***	0.015 ***
	(0.000)	(0.000)
ESGf	−0.005 ***	−0.003 ***
	(0.000)	(0.000)
ESGf*Post	−0.002 ***	−0.002 ***
	(0.000)	(0.000)
Size		−0.001 ***
		(0.000)
Lev		−0.000
		(0.000)
TobinQ		0.002 ***
		(0.000)
Cash		−0.027 ***
		(0.003)
Constant term	0.030 ***	0.052 ***
	(0.000)	(0.002)
*N*	38,638	38,638
*R* ^2^	0.122	0.142

Note: *, ** and *** represent significant at the level of 10%, 5% and 1% respectively.
